# VALIDATING THE PREFERENCES ASSESSMENT TOOL FOR NURSING HOME RESIDENTS USING ITEM RESPONSE THEORY

**DOI:** 10.1093/geroni/igz038.1238

**Published:** 2019-11-08

**Authors:** Yinfei Duan, Weiwen Ng, Tetyana P Shippee

**Affiliations:** 1 School of Nursing, University of Minnesota, Minneapolis, Minnesota, United States; 2 University of Minnesota, Minneapolis, Minnesota, United States

## Abstract

The Preference Assessment Tool (PAT), part of the Minimum Data Set (MDS), assesses residents’ preferences to enable preference-based care in nursing homes (NHs). The two PAT sections including daily routine preferences and activity preferences are assumed to measure autonomy and meaningful activities as the underlying constructs associated with residents’ psychosocial needs. Yet, the validity of this assumption has not been tested. This study examines PAT’s construct validity using item response theory. We fitted graded response models to the two PAT sections using 2017 MDS annual assessments of 8,829 long-stay residents in 291 Minnesota NHs. Most items discriminated well between residents who had at a low versus high intensity of these two types of psychosocial needs (i.e. have discrimination parameters > 1). Two daily routine preference items (family’s involvement in care planning, and having snacks), and three activity preference items (having pets, keeping up with news, and reading) had poor discrimination in measuring autonomy and meaningful activities respectively. Overall, the PAT appeared to be more sensitive in measuring the lower middle range of the two constructs. We estimated the correlation between the two constructs as 0.65. In conclusion, the PAT performs fairly well in measuring the two types of psychosocial needs for NH residents. Nevertheless, more items are needed to capture a broader range of psychosocial needs beyond autonomy or basic leisure activities. The findings of this study brought attention to the utility of the PAT in measuring residents’ psychosocial needs and in guiding resident-centered care.

